# Birmingham Infirmary

**Published:** 1830-10-01

**Authors:** 


### BIRMINGHAM EYE INFIRMARY.

MR. MIDDLEMORE, assistant-surgeon to this institution, has published, in the Midland Reporter, a quarterly report of the cases treated at the establishment during the last three months.* The great majority have consisted, of course, of the different varieties of ophthalmia, opacities of the cornea, iritis, amaurosis, and tinea. Appended to the numerical report are some observations on iritis and amaurosis, by Mr. Middlemore, which are deserving of attention.

### I. IRITIS.

Mr. Middlemore has been experimenting with the spiritus terebinthinæ in this disease, and the results appear to be confirmatory of the observations of Mr. Carmichael and Mr. Guthrie. He has given it with great advantage to patients who, from delicacy or peculiarity of constitution, are unable to take mercury to the production of salivation. If, too, the acute symptoms have been relieved by mercury, but a chronic disorganizing inflammation remain, Mr. Middlemore would strongly recommend the exhibition of a drachm of turpentine two or three times a day. He would urgently advise the early employment of turpentine in inflammation of the choroid and retina.

"Strumous iritis is a disease somewhat uniform in its appearance and history, obstinate in duration, and very little influenced by the modes of treatment usually recommended for its cure. The individuals most obnoxious to its attacks, are children, between the ages of six and eighteen, of a delicate constitution, fair complexion, light hair, and blue eyes; as soon as the disease has become established, the cornea assumes a misty appearance, patches of red vessels are frequently seen upon its edge; there is a zone of pink vessels around the cornea in various situations; the patient is troubled with profuse lachrymation, and great intolerance of light; the iris is almost inactive, and vision is considerably impaired; the eye-brow appears to project considerably before the eye; the muscles (if one eye only be affected,) become thicker and stronger than those of the opposite side, from their powerful contraction to exclude the light, and give to the countenance a distorted appearance; the pulse is generally quick and irritable; the appetite uncertain; the secretions unhealthy; the skin dry and harsh, but variable in temperature, the heat of the scalp being oppressively great, whilst the extremities are often chilled with cold. It would be trifling to enumerate the various plans of treatment recommended for the cure of this troublesome disease; no 'one plan' will succeed; a treatment directed to the circumstances of each particular case, will always be necessary; for a delicate child, I should advise small doses of the hydrargyrus c. creta every other morning; a grain of quinine twice a day; every other evening a warm bath, and should the skin be harsh and dry, notwithstanding its employment, friction with the hand, or a soft brush should be used, and on no account should suitable clothing, with a view to maintain perfect warmth of the surface, be neglected; a small issue in the arm; mo-

---

* Mid. Med. and Surg. Reporter, No. VIII. May, 1830.

derate (not fatiguing) exercise must not be omitted, and perhaps riding on horseback surpasses in excellence all other modes of taking it; without specifying any particular articles of diet, I may say in the general, that it should be of a light and nutritious character, animal food being allowed only every other day."

II. Amaurosis.

The following remarks are chiefly on the exhibition of strychnine in that formidable malady amaurosis. It is a remedy which is now in some vogue, not only with oculists but with physicians also, in the palsy-cases of middle and advanced age, whether the affection be of the retina or of one side of the body, of a solitary muscle or a large portion of the material frame. The very nature of these cases, we mean their connexion with advanced life, and their dependence on the wear and tear of the machine, precludes the hope of any remedy or class of remedies proving extensively and permanently useful. Medea's cauldron would suit such cases, but short of the youth-restoring drug of the enchantress, there is nothing, alas! that will again give elasticity to the withered sinew: plumpness and vigour to the wasted muscle; or the finely-tuned sensibilities of adolescence to the palsied nerve. Nevertheless, there are, undoubtedly, some cases of palsy and amaurosis, we fear they are fewer than is imagined, that are benefited by the exhibition of the strychnine or stimulating medicines of that class. The discrimination of these must be a matter of experiment and experience. We will give the results of Mr. Middlemore's.

"If a patient has overworked the eye by long-continued action, confined to the inspection of objects of the same colour and description, an enfeebled condition of retina (just as we produce an exhausted state of muscle by over-exertion) will take place. If a man subject his eye to an unnatural stimulus, by looking for many hours daily at bright substances of the same or nearly the same colour—or to sudden transitions from an artificial glare to comparative darkness (as miners)—or to a diminished stimulus, as by working in dark rooms, or places imperfectly supplied with light—or to any cause allowing the visual textures of the eye to remain, for a long period, in a state of inactivity, as takes place where large opacities of the cornea, and fully-formed cataract exists, the power of the retina will be partially destroyed—its susceptibility to the stimulus of light diminished; but in none of these cases will there be found any structural change in the retina or the optic nerve, any congestion of vessels, or any discoverable alteration from a healthy and natural condition; nor will the system, in all probability, be found affected; no altered state of health sufficient to account for the dimness of vision, will be found to exist. At some kinds of employment it is necessary for the individual to work with the head bent forwards, declining, or the body so distorted as to favour the too liberal flow to the eye, and retard its return; inducing what is termed congestion; a distended state of vessels, unfavourable to free and active circulation; a condition of eye which is also frequently induced by the investigation of minute objects by the aid of powerful glasses. Loss or diminution of the power of vision sometimes comes on from certain causes which diminish the vigour of the system generally—as for instance: after profuse salivation, long continued suckling, menorrhagia, &c. In all these cases, I believe, the strychnine is calculated to produce great and permanent advantage, in combination, of course, with other remedies suited to the particular exigencies of the case—for example: if the retina be weakened in consequence of diminished vigour of the system, remedies adapted to strengthen the system, and a removal of the cause enfeebling it, might be joined to the local application of the remedy in question. But the power of the retina will not always return with the returning strength of the system; in such cases the strychnine is singularly valuable, producing, with wonderful rapidity, the restoration of the organ of vision. Strychnine given internally does not produce the same beneficial effect upon the retina, as when applied externally. The mode of using it is already before the profession. After having tried it in a variety of ways, and in different situations, I have not been able to discover a better method than that of blistering the skin above the eye-brow, and, after having carefully removed the cuticle, I sprinkle the powder upon the raw surface, taking care to pass a spatula upon the part so sprinkled, to secure it against removal and insure its absorption; a piece of linen (not greased) should afterwards be bound upon the part. The quantity with which I generally commence is the twelfth of a grain upon each side, daily augmenting the quantity, as the patient is enabled to bear it, until it amounts to the ⅔ of a grain upon each blistered surface. Its first effects are—slight pain in the head, increased power of vision, and severe smarting pain of the part upon which it is applied. Some patients cannot bear its application; others require great care, and a very gradual augmentation of the quantity to enable them to bear it; whilst others will admit of its application without experiencing any other uneasiness than what arises from its action upon the sore. It is not necessary, I presume, to detail cases in support of my views; such a plan would greatly extend my observations, which I have been studiously anxious to limit.—I will now, for a short time, draw the attention of your readers to those cases in which the employment of this remedy would be useless or injurious. If the amaurosis be dependent on any morbid condition of the brain: any alteration of the bony structure; any tumour or other substance pressing upon the optic nerve, the effects of former inflammation, such as opake deposition or partial disorganization, the effusion of blood or morbid growths, the enlargement of the vitreous or displacement of the crystalline humour, producing pressure upon the retina; a varicose state of vessels, as a consequence of distention so continued as to impair their tonic and elastic properties; inflammation of, or disease of, those parts incased by, or anterior to, the retina,—no benefit could be expected to result from the use of strychnine; but, on the contrary, in many of the cases, material injury might succeed its employment."

We certainly have not seen that benefit from strychnine in general palsy, which some authors would lead us to believe was frequently obtained by it. Mr. Guthrie has been testing its powers in amaurosis, but without any great success. Mr. G. has laid the results before the profession.

